# Tackling Complexity in High Performance Computing Applications

**DOI:** 10.1007/s10766-016-0422-9

**Published:** 2016-04-16

**Authors:** J. Darlington, A. J. Field, L. Hakim

**Affiliations:** Department of Computing, Imperial College London, London, UK

**Keywords:** High performance computing, Application development frameworks, Functional workflows, Constraint solving

## Abstract

We present a software framework that supports the specification of user-definable configuration options in HPC applications independently of the application code itself. Such options include model parameter values, the selection of numerical algorithm, target platform etc. and additional *constraints* that prevent invalid combinations of options from being made. Such constraints, which are capable of describing complex cross-domain dependencies, are often crucial to the correct functioning of the application and are typically either completely absent from the code or a hard to recover from it. The framework uses a combination of functional workflows and constraint solvers. Application workflows are built from a combination of functional components: higher-order co-ordination forms and first-order data processing components which can be either concrete or abstract, i.e. without a specified implementation at the outset. A repository provides alternative implementations for these abstract components. A constraint solver, written in Prolog, guides a user in making valid choices of parameters, implementations, machines etc. for any given context. Partial designs can be stored and shared providing a systematic means of handling application use and maintenance. We describe our methodology and illustrate its application in two classes of application: a data intensive commercial video transcoding example and a numerically intensive incompressible Navier–Stokes solver.

## Introduction

Despite many advances it still remains the case that developing, using and maintaining complex high performance codes is a laborious manual activity, requiring the expenditure of many skilled person hours. The basic code-centric application development model has remained largely unaltered despite some changes in the programming languages used. This means that developing, maintaining and using HPC codes, which are generally complex mathematical and software objects, can be difficult. The field still often relies on heroic personal endeavours to make progress and the complexity of the technology means that there are considerable barriers to entry for many potential users who could otherwise benefit from the capabilities available. Cloud computing has made large-scale computational resources available to many people who otherwise would not have access to them. What is needed is corresponding developments on the software side to make HPC methods equally accessible and usable.

If one examines the structure of the HPC eco-system one can begin to see what may be the underlying causes of this problem. All players in the HPC application stack (end-users, method developers, processor designers, machine architects and facility providers) quite rightly want to push their activity to the limit. So, end-users want to model ever more complex systems to finer degrees of fidelity, method developers introduce ever more sophisticated but complex solvers (e.g. spectral/hp element methods), processor designers, in the search for ever more flops, resort to complex and extreme chip architectures (e.g. many-core, GPU) and machine architects and facility providers seek to develop and operate ever more powerful infrastructures (e.g. large-scale distributed clusters or clouds).

Of course, this is all to the good and the only way progress can be made but it does introduce much complexity. Within each sector there are many alternatives or choices to be made. For the user, these comprise issues such as what is the science to be modelled, what scale or fidelity to be attempted and at what cost (or energy use). For numerical methods, for example, there are issues such as which solver to use, what time integration scheme to incorporate and what polynomial order to evaluate. For processors there is the degree of concurrency to support, synchronisation and cache behaviour and for machines whether to use servers, clusters or clouds and how many processors to use.

Furthermore, configuring an application to do what the user wants is often extremely complex because of subtle dependencies between the various configuration parameters that the developer has chosen to expose. For example in a fluid dynamics solver the choice of numerical algorithm may influence the problem specification, and vice versa, e.g. a low-order polynomial problem may require the construction and solution of a global matrix problem whereas a higher-order problem may be best solved using an elemental approach. Note that other types of dependency may also arise, e.g. the application may depend on a particular version of a library being installed and/or on the user having relevant licences to use a particular piece of software upon which the software depends.

The key point, we feel, is that when an application is constructed, all these choices compound and the decisions that are taken are often largely in the head of the developer. The net result, and end-point of these decisions, is code. However, code expressed in a conventional programming language is incapable of explicitly recording these decisions nor are these decisions recoverable from the code. Thus knowledge is lost and the decision structure, that led to the code being as it is, is not available when the code is used, modified or developed. This, to our mind, is one of the reasons HPC remains a difficult technology to use by people not specialist in all these areas and also means that when changes are made to the code they are often done in an ad-hoc manner, which usually means that code structure and usability deteriorates over time adversely affecting usability and maintenance (sustainability) of these codes. It is for these reasons that we believe that the issues concerning HPC software cannot be resolved solely by improving the programming practices employed. Our thesis is that these problems lie not with the programming languages per-se but with the way they are used. We therefore need to develop frameworks that are capable of capturing or recording the decisions taken and making this knowledge available for effective use.

In this paper we describe such a framework for application code development that is capable of capturing key decisions taken during application development and making this knowledge available to support both end-use and the long-term development and maintenance of these codes. Within this framework an application is defined by a workflow that composes software components, including *coordination forms*, pre-defined constants and model/configuration parameters (free variables). The coordination forms [9], often referred to as *skeletons*, are higher-order functions that abstract some control orchestration pattern, for example map, reduce, filter, farm, pipe etc. The framework embodies several key ideas:

- Nodes within the workflow may be defined to be *abstract*, which means that they define functionality without specifying an implementation. A workflow can thus be instantiated by specifying concrete implementations for the abstract methods in addition to instantiating traditional model parameters.
- By archiving workflows and their instantiations as they evolve from abstract to concrete, we naturally expose the provenance of a particular ‘build’ of the application. The workflow defines what computation *should* be performed, whilst a specific profile defines *how* it should be performed. Indeed, it is straightforward to revert back to earlier workflows and then construct different concrete implementations to those made originally.
- Because the instantiation options are explicitly identified within the workflow it is possible to specify additional *constraints* that describe the dependencies between various component implementations and parameters. By automatically invoking a constraint solver at each parameterisation step we can ensure that it is impossible to construct a concrete workflow that is internally inconsistent.

Our approach combines the expressive power of high-level workflows, and logic programming, which we use to specify and manage constraints. Workflows are ubiquitous in high-performance computing, of course, but the idea of allowing workflow components to be abstract provides a powerful vehicle for exposing the component implementation choices that need to be made in order to construct a valid executable.

The key idea, and the main contribution of this paper, is the use of constraints to determine valid instantiations of a workflow. This is much easier to do when the configuration options are made explicit as part of the workflow. Referring back to the fluid dynamics example above, we may wish to forbid the selection of a global matrix algorithm for high-order polynomial problems, for example. The polynomial order in this case would be captured as a workflow parameter and the type of numerical solver to use would be captured as a specific instance of an abstract method, solve, for example.

We present our workflow-based development framework in Sect. 2 and details of the workflow engine and constraint solver in Sects. 2.1 and 2.2. In order to illustrate the ideas in practice we develop a simple video transcoding workflow in Sect. 3.1. In particular this shows how component implementation selection and the constraint solver interact in order to produce a consistent workflow instantiation. In Sect. 3.2 we show how the configuration options in a complex incompressible Navier–Stokes solver can be captured explicitly as constraints over component implementations and parameters within a workflow that captures the top-level structure of the solver. In Sect. 5 we discuss various ways in which our ideas can be developed, with particular reference to transformation-based workflow optimisation and HPC application provenance.

## Framework

**Fig. 1:**
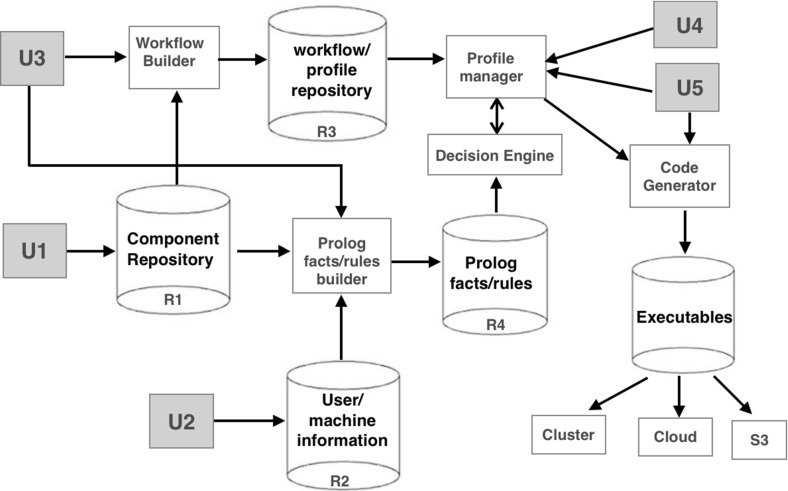
Framework

Figure 1 provides a schematic overview of our framework. This is centred around a workflow script that describes a specific computational problem (see Sect. 2.1). The ultimate objective is to instantiate each of the parameters of the workflow, including concrete implementations of abstract components, to form a concrete instance that can be executed on a given target platform. The constraint solver (Sect. 2.2) is used to ensure that a particular workflow parameterisation is internally consistent. The decision engine together with the Prolog facts and rules, shown in Fig. 1, form the constraint solver.

The internal organisation of the framework is best described by highlighting the role of the five main types of user shown in Fig. 1:

- Component developers (U1) construct application-specific codes that may be applicable to several problem domains, e.g. a video transcoder or finite element solver. They may also be responsible for developing new coordination forms akin to the familiar forms such as map, reduce etc. These building-block components, including associated metadata that documents the relationship between the abstract forms and their realisations, are stored in a component repository (R1). In many cases concrete component implementations may be provided in a pre-existing library in which case the repository will contain a reference to the relevant library in addition to the metadata that identifies its abstract equivalent.
- Administrators (U2) maintain information about users, organisations and resources. This information will typically determine indirectly what privileges each user has, including, for example, the hardware platforms and licences that the user, or the institution they work for, has available. This administrative data is collected together in a separate repository (R2).
- Workflow developers (U3) are responsible for the construction of workflows that bring together the various components and coordination forms to solve a particular computational problem. Workflows in the framework are written in Python syntax (see Sect. 2.1), but may refer to abstract methods and parameters, all of which must be instantiated before the workflow can be executed.
- Profile developers (U4) are responsible for (partially) parameterising a workflow, e.g. choosing concrete implementations for particular abstract components. A specific parameterisation of a workflow is referred to as a *profile*. Central to the process of profile development is a decision engine that ensures that each profile is internally consistent. In particular, users are prevented from instantiating parameters or abstract methods that are inconsistent with a given set of constraints (Sect. 2.2). The application-specific rules that the decision engine relies on are specified by the workflow developer (U3 above); the assumption is that it is they who have the expert domain knowledge required to formulate such rules. In the present implementation these rules are expressed directly as Prolog clauses that are stored through to a fact/rule repository (R4) via a fact/rule builder. In future we envisage that more user-friendly formalisms or tools will be used to specify such constraints, in which case their Prolog equivalents will be auto-generated. Note that the various Prolog facts referred to by these rules *are* generated automatically by a fact builder using information extracted from the user repository (R2). The final profile(s) generated from a workflow will typically have a few remaining parameters to be set, e.g. input/output file locations, and these will be filled in by the end users. The various workflows and profiles are stored in a repository (R3).
- The end users (U5) may have little or no knowledge of the detailed computation described by the workflow. They are interested in running a workflow and their role is typically to provide the final set of application parameters, e.g. the input/output files, needed to fully instantiate that workflow. The use of profiles and constraints serves to protect the user from making the sort of ‘obvious’ mistakes that the domain expert typically knows to avoid, but which so often go unchecked in the application itself. A fully parameterised workflow is in principle an executable Python script although in practice the final executable also contains wrappers e.g. for moving data to/from the execution platform where the workflow will run.

### Workflow Engine

The framework abstracts three aspects of general computation: control, data processing and storage. Control is specified using coordination forms, such as map, reduce, filter, farm, pipe etc.

We allow the definition and use of an extensible set of coordination forms, although at any one time a user will be using a fixed set of such forms. We abstract data processing methods as components encapsulated as first-order functions. These will generally be encapsulated methods from the application domain.

The implementation we have built uses Python syntax for the workflow scripting language. We use Python’s own parser module which provides the necessary tools for identifying workflow parameters and abstract methods, which would otherwise be treated as undefined variables. In the current prototype we do not restrict the language in any way. However, in order to extend the framework to include features such as meaning–preserving program transformation, immutable data etc. it would make sense to restrict the language to only pure functions, or at least single-assignment semantics. That is left for future work. Note that we consciously refrain from treating workflows as graphical objects as visual representations quickly become cumbersome as the complexity of the workflow increases. Also, simple static data flow “pipelines” are incapable of capturing the dynamic computational patterns of general purpose control structures.

### Constraints

Each workflow, together with the implementations and machines available, gives rise to what we call a *Decision Space*: all feasible realisations of the workflow and mappings to the machines available. How this Decision Space is navigated is at the heart of our methodology. Navigation is realised as an interaction between an option selection process (managed by a user interface within the framework) and a Prolog-based decision engine operating in the background. Thus the essence of our methodology is not creating a constraint solver which limits options per se, but it is the idea of connecting the several parts of a framework which allows several users to take part in different ways, from creating a workflow and introducing components to navigating through an interface to obtain an output.

In our present implementation the information in the component repository is used to populate a Prolog database encoding the mappings between abstract functions and their possible implementations. The database is also augmented (automatically) with additional information required by the constraint rules, for example users, their affiliations, software packages and licenses, available machines etc. Some examples of the use of constraints is given in Sects. 3 and 3.2 below.

The use of workflows to define transcoding tasks enables various players to play a part in producing a complete solution. The developed prototype has been used in various real-world use cases.

Examples in industrial use have been implemented where a video transcoding task has been implemented where the developers built the components and coordination forms needed to transcode a video, as well as constructed the Prolog constraints for use in the decision engine. The end users were then able to navigate through the user interface by selecting choices for entities such as organisation and software licenses as well as selecting the input video desired for the job. The end users do not have any connection with the code, they only make selections of parameters they want to use in order to transcode the video. However, the user interface will support all sets of users such that they are all interfacing in the same abstract space.

## Application Examples

We observe that there are broadly two classes of high performance computing applications, each with their own software methodologies and working practices: numeric and data intensive computations. Numerical applications (such as PDE solving) tend to have monolithic code bases written in high-level languages such as Fortran, C or C++. Furthermore effective use of these codes often requires close collaboration between the method developers and end users. Data intensive applications, on the other hand, tend to be built out of separate composable components and programmed using scripting languages such as Python. In these areas there is often a more established tool producing community separated from end users and connected via the development and distribution of component libraries, e.g. bioinformatics.

Both areas, however, share the underlying characteristic that there are many choices or decisions to be made that govern the correct and efficient use of these applications.

In this paper we will first look at the application of our methodology to a typical data intensive application, processing or transcoding of media (video) files, and then to a PDE application, that solves the incompressible Navier Stokes equations using a spectral finite element method.

### Data Intensive Application Video Processing

We now show how the framework can be used to construct a simple video transcoding workflow that performs the following steps:

1. Read an input video (inputVideo)
2. Crop the video to a specified aspect ratio
3. Add subtitles (subtitleList) in *n* different languages to the cropped video, generating *n* output videos
4. Concatenate the *n* videos into a single output video

**Fig. 2:**
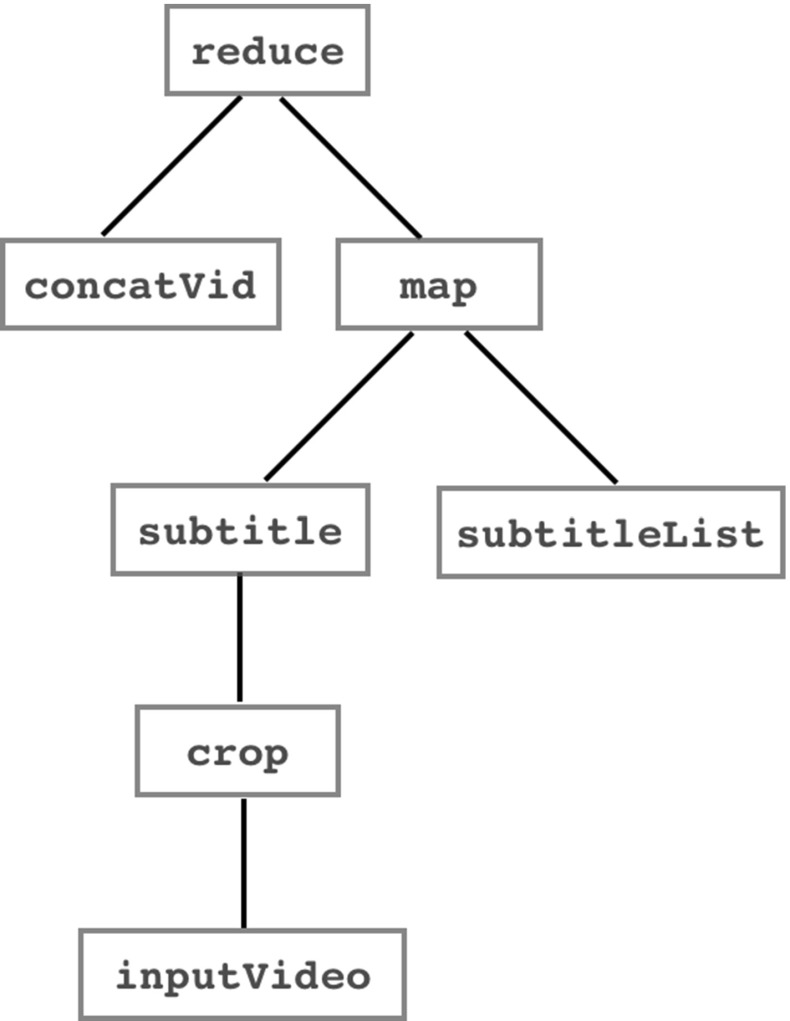
Workflow in abstract functions illustrated as a tree

The workflow is illustrated diagrammatically in Fig. 2 and can be implemented by the following Python workflow function: 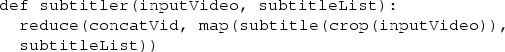

The script makes use of the abstract functions map, reduce, subtitle, crop, concatVid which specify *what* operation should be performed at each step without committing to *how* that operation should be implemented. map and reduce here are generic coordination forms and the other three are functions specific to the domain of video transcoding.

Note that an invocation of the abstract function subtitle requires both an input video and a subtitle file. Here we *partially apply* the function to a single cropped input video. The abstract map function map supplies the partially-applied crop function with a succession of arguments from the list of (*n*) subtitle files subtitleList.

Why do we refer to abstract, rather than concrete, functions? This is because we may want to be able to implement the same generic workflow differently depending on the resources available at the point of execution. For example, the choice of video transcoding function might depend on the transcoding libraries available to a particular user of the workflow: we may prefer to use a proprietary library in preference to an open-source library, if it is available, for reasons of efficiency or image quality, for example. Similarly there might be both sequential and parallel implementations of the various abstract functions over collections (e.g. lists) and the user may prefer to use the parallel versions of those functions if they have a multi-core or cloud computing platform available. Here, parallelism would facilitate the different language subtitles to be added concurrently, for example.

Before an abstract workflow such as this can be executed it is necessary to specify both the values of the free variables (parameters) and the concrete implementations of the various abstract functions referred to. Note that these, as yet unspecified attributes, are identified when the script is parsed. Traditionally, workflow parameters would be defined by the user, but the idea here is to employ a separate constraint system to ensure that the user can only select valid combinations of parameters and component implementations.

#### Constraint Solving

To illustrate how constraint solving can be used to explore the Decision Space generated by an abstract workflow, we will now define some constraints that are universally applicable to all workflows and show how they can be applied in the context of the video transcoding example above. Specifically, we wish to ensure that the implementation options and parameters chosen by the user are consistent with the resources that they have at their disposal. The rules are stated informally as follows:

1. A user can access a machine if they work for an organisation that owns that machine.
2. A component implementation can be run by a user on a given machine if the user has access to the machine and both the machine and implementation have the same execution mode (e.g. sequential or parallel).
3. A component implementation can be used to run a specified abstract function if that implementation has been predefined to be appropriate for that specific abstract function.
4. A transcoding library can be used to run a component if the user running the concrete function works at an organisation that has a license to use that library.

These constraints represent the additional information that would not normally be part of the application’s code base.

In order to implement these constraints the workflow developer needs to encode them, here as Prolog clauses. These clauses will refer to additional Prolog facts that are generated automatically from the various repositories shown in Fig. 1. For example, the various implementations of the abstract coordination forms (map, reduce etc.) and application-specific abstract components can be extracted from the component repository (R1 in Fig. 1). An example might be the abstract video processing function crop, which will be stored alongside its various available implementations, together with additional information (meta-data) about their required licenses, execution modes etc.

**Fig. 3:**
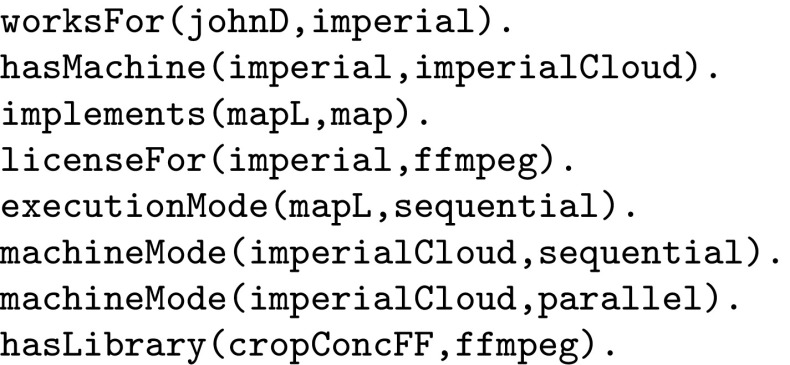
Examples of automatically generated facts

Figure 3 shows an example of some facts generated from the component and administration repositories (R1 and R2 in Fig. 1). Here, johnD, imperial, ffmpeg and imperialCloud are examples of a given user, organisation, transcoding class and machine respectively. Also, mapL is an implementation of the abstract function map, and sequential/parallel represents the ability to run a job in sequential/parallel mode. Note that, variable names have an upper-case initial letter while constants have a lower-case initial letter. Crucially, notice that the Prolog clauses here may refer to variables (abstract methods and parameters) that are referenceable from the workflow, such as map.

**Fig. 4:**
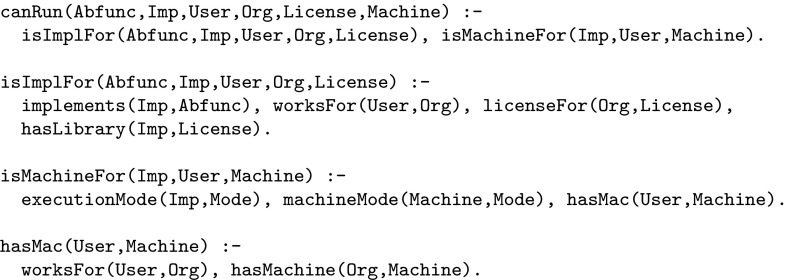
Constraint clauses

The Prolog clauses that implement the constraint rules above are shown in Fig. 4. Notice that these refer to the auto-generated facts in Fig. 3. Once the database of Prolog facts and constraint clauses has been set up they can be referred to as part of any workflow instantiation process.^1^ In our prototype we have implemented a decision engine for controlling this instantiation process: each time the user makes a selection in the user interface, the Prolog constraint solver is invoked. The result of each such invocation is a set of valid settings, i.e. feasible solutions to the Prolog goal, for each of the remaining implementation/parameter settings in the workflow. By this form of interaction we ensure that it is impossible to specify a workflow instantiation that is inconsistent with the constraints.

We note that the current prototype framework requires a developer to enter the Prolog constraint rules shown in Fig. 4 ‘by hand’. This is arguably quite cumbersome and we would instead prefer to generate such rules from, e.g. a suitably-defined domain-specific language (DSL) or library for specifying application-specific constraints. That is the subject of ongoing work. This is, however, the only Prolog code that has to be produced manually by the developer.

#### Profiles

**Fig. 5:**
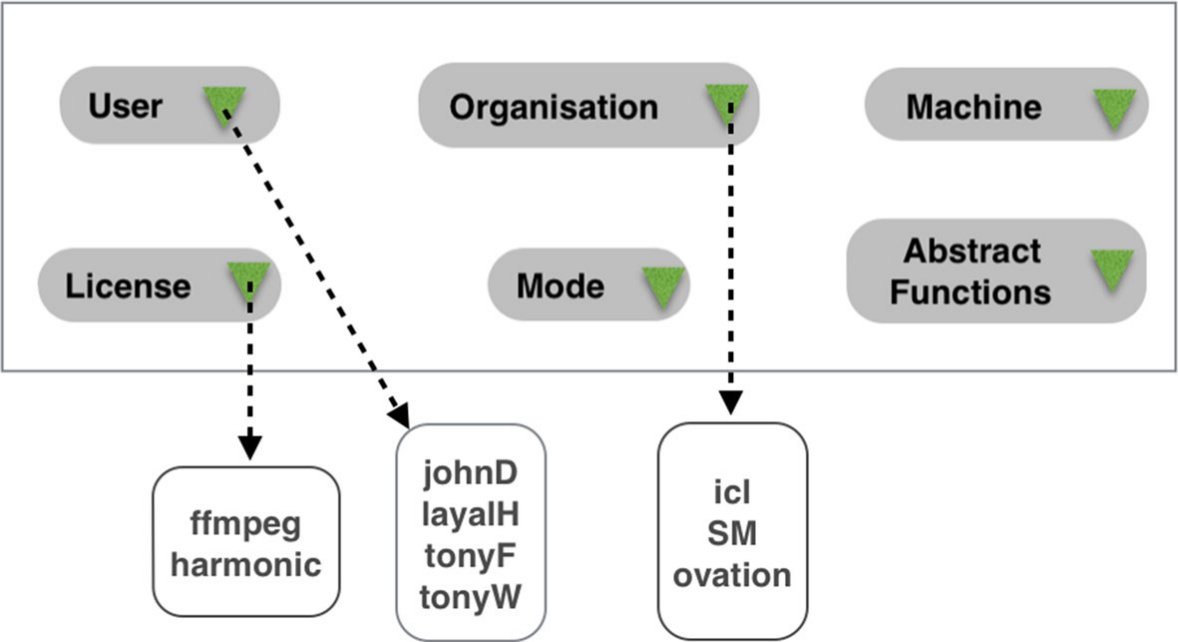
Profile example

Any abstract workflow, together with the Prolog database, generates what we call a template or *profile*—a set of all the options available to the user. This is presented to the user through a GUI comprising selections for all the available options (Fig. 5). Here, the Mode provides a very simple distinction between execution modes that are here assumed to be either sequential or parallel, by way of illustration. Furthermore, the Abstract Functions drop-down box in Fig. 5 gives a full list of abstract functions which once selected can direct the user to another drop-down box which gives a list of options for the component implementations that correspond to the chosen abstract functions.

**Fig. 6:**
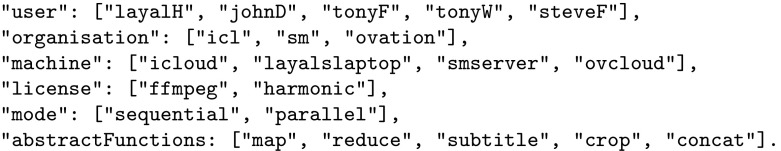
Full dictionary for the workflow

We will use *dictionary* to refer to the data structure that mediates between the profile and the user interface. The workflow automatically produces the dictionary illustrated in Fig. 6.

**Fig. 7:**
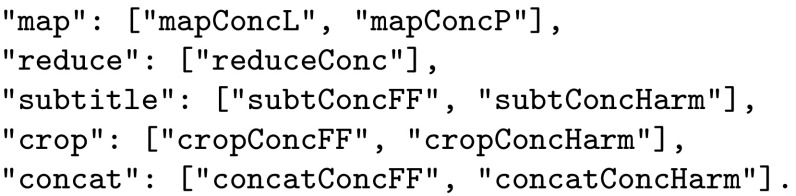
Example of a dictionary for the abstract functions

In order to invoke the constraint solver the Decision Space manager inspects the current workflow identifying the remaining abstract methods and unspecified parameters. From this it constructs a Prolog call which is passed to the constraint solver (we use PySWIP to read and connect the SWI Prolog [2] code within Python). Referring back to Fig. 2 the workflow contains the five abstract functions map, reduce, subtitle, crop, and concat. Each of these will need to be instantiated to a concrete implementation and the choice of implementation will be subject to the constraints being satisfied. Given in Fig. 7 is an example of a dictionary for a set of abstract functions; this is generated automatically from the metadata stored in the component repository (Fig. 1).

**Fig. 8:**
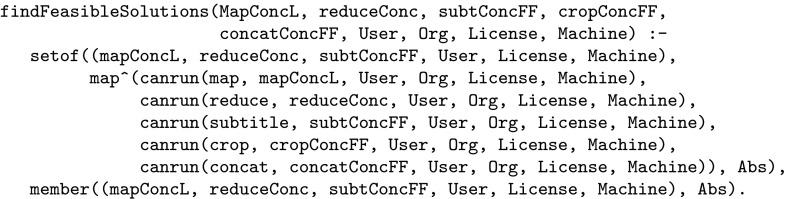
Example of a top-level call

Here, for example, we want to prevent the user from selecting an implementation for which they have no licence, in this case using the canRun predicate above. To this effect the framework builds and executes dynamically the top-level call findFeasibleSolutions shown in Fig. 8. This uses the Prolog built-in predicates setof and member to remove duplicate solutions from the final set of output solutions.

**Fig. 9:**
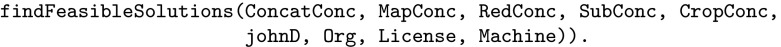
Top-level call after a selection has been made

The key advantage of using Prolog in this setting is that the order in which the user chooses to instantiate the workflow parameters and concrete function implementations is unimportant. For example in an invocation of the relation canRun(Abfunc,Imp,User,Org,License,Machine) the user may first choose to identify the licence(s) that they wish to exploit and this will restrict the set of abstract functions (Abfunc), implementations (Imp) and machines (Machine) that they can subsequently select. Alternatively, they may select an abstract function to instantiate in which case the constraint solver will restrict the choices of implementation and machine, together with any licences that are required to implement the function. To illustrate this, suppose we select the user to to be johnD. This is presented to the Prolog engine, and the corresponding top level call originally shown in Fig. 8 is given in Fig. 9.

**Fig. 10:**
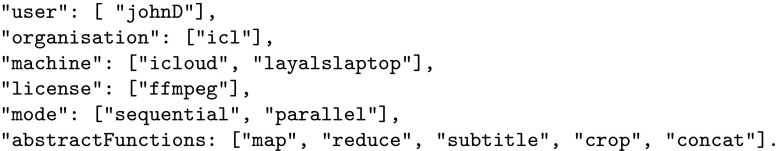
Updated dictionary after selecting user johnD

**Fig. 11:**
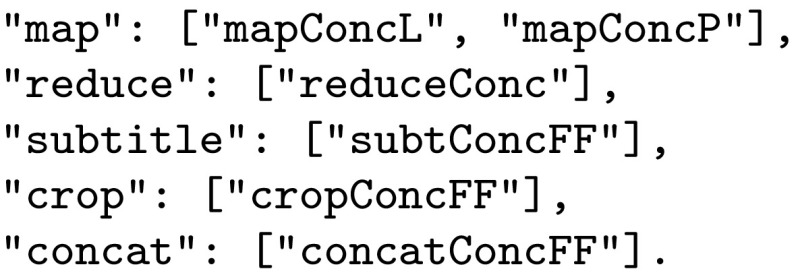
Updated dictionary for the abstract functions

**Fig. 12:**
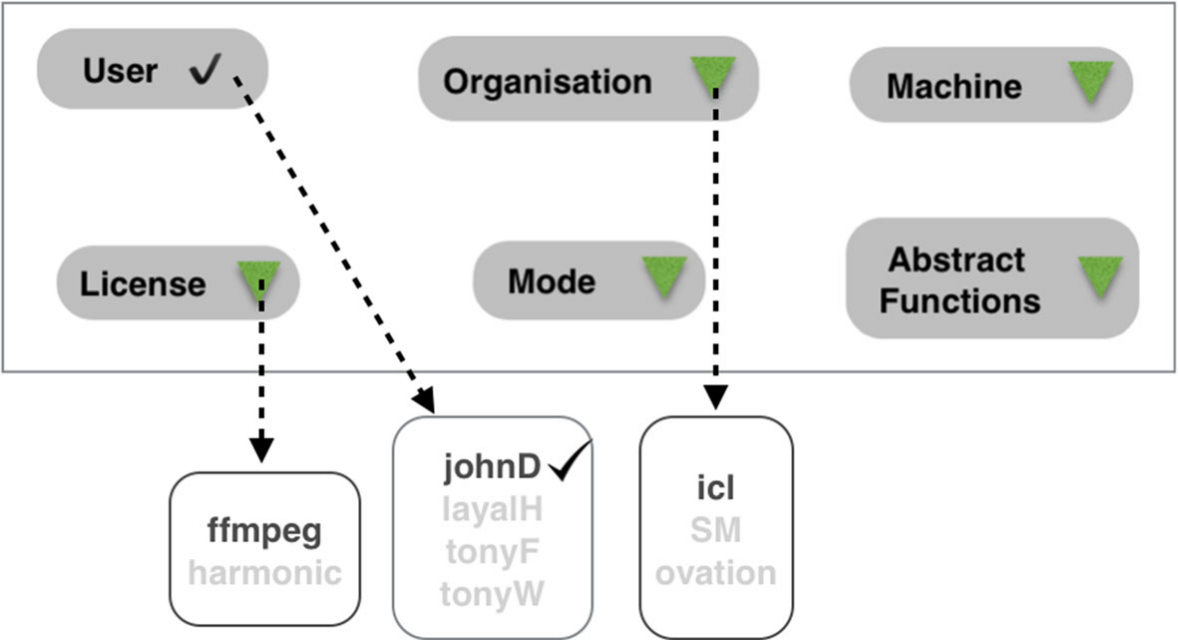
Updated profile

Each selection in a profile represents a move in the Decision Space. The Prolog generates all feasible solutions still consistent with this selection. The set of all feasible solutions returned in this example is shown in Figs. 10 and 11 and the UI is updated as shown in Fig. 12.

This process continues with the user making selections and the Prolog updating the Decision Space until we have a completely instantiated profile (or profiles). These represent executable realisations of the workflow that can be selected and executed.

Although we do not show the output from workflows such as the one described, we highlight the fact that the prototype framework is fully functional and is being used to define various commonly-occurring transcoding workflows, e.g. video subtitling and video ‘stitching’, which involves the packaging of broadcast video (e.g. a TV advert) with pre- and post-content whose exact format is specific to the country/region to where the video will be shipped.

### Incompressible Navier–Stokes

In order to illustrate how constraints can be exploited in a more sophisticated numerical modelling application, we now consider the Nektar++ spectral finite element code for solving incompressible fluid flow problems [5]. The present version of this code is a C++ executable that can be configured for a variety of different problem types and physical geometries by providing a parametrisation file as input. Generating a valid combination of parameters is not easy and this is where we wish to exercise the idea of using constraints. In this case there is no top-level workflow, just a monolithic code base. Nevertheless we can still make explicit the decisions or choices required for correct and efficient use of this code and we focus on the manual construction of Prolog facts and constraints, guided by the structure of the model parameter file.

**Fig. 13:**
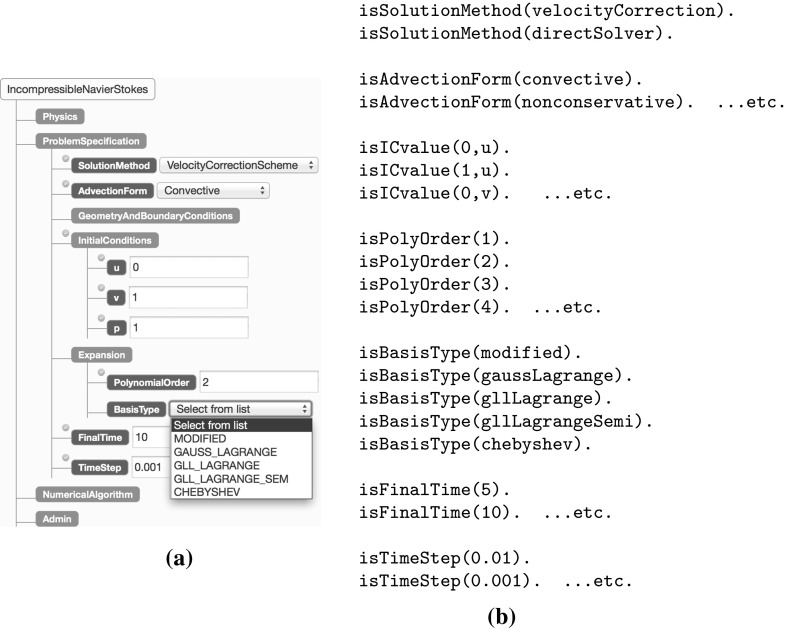
Partial Nekkloud decision trees and Prolog equivalent. **a** Nekkloud screenshot, **b** equivalent Prolog relations

In order to illustrate how Nektar++ is parameterised Fig. 13a shows a screenshot of part of the Nekkloud system [8] which is a separate utility that can be used to configure a Nektar++ model instance. Note that there are four parameter sets (Physics, Problem Specification, Numerical Algorithm and Admin), but only the Problem Specification tab is shown expanded. Note that the Nekkloud screenshot is included purely to help clarify the structure of the the Nektar++ parametrisation and corresponding Prolog code.

The principle we follow is that each configurable Nektar++ parameter class is captured as a Prolog relation with the valid parameter values being instances of that relation. To simplify the Prolog code, we restrict each parameter to one of a small number of predefined settings. For the Basis Type, for example, the valid settings range from Modified to Chebyshev, as shown in the Nekkloud pull-down menu in Fig. 13a; for u, v and p we allow values of 0 or 1 for the purposes of the example. A subset of the Prolog code corresponding to the Nektar++ Problem Specification parameters is shown in Fig. 13b.

**Fig. 14:**
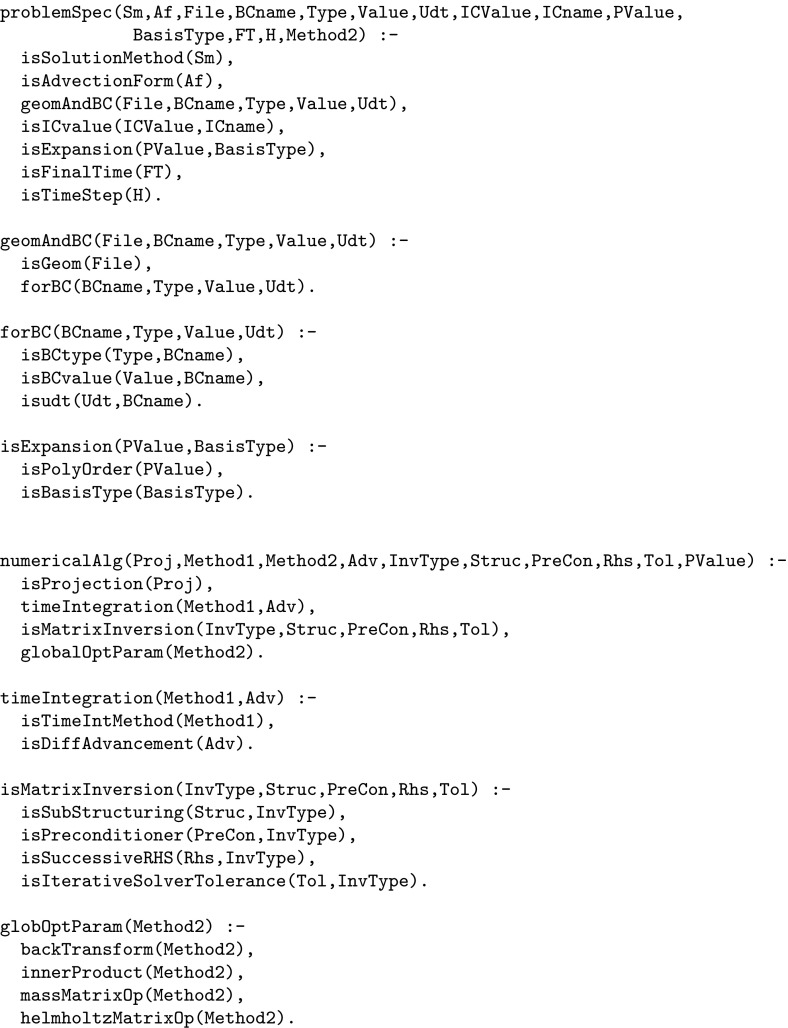
The problemSpec and numericalAlg parameter classes for Nektar++

**Fig. 15:**
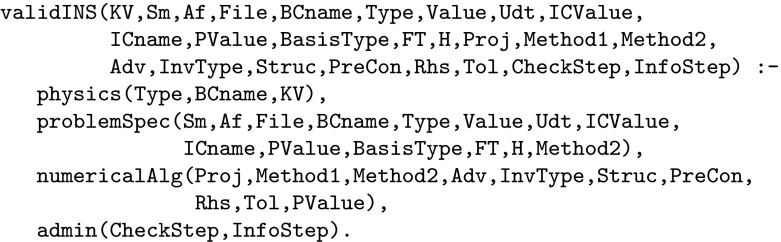
Top-level call for the Incompressible Navier–Stokes problem

As in the transcoding example above we proceed by specifying a top-level Prolog call that combines all of the Nektar++ parameters into a single relation. This is defined in terms of four sub-relations, reflecting the four parameter subsets defined by Nektar++ and implemented within the Nekkloud interface (Fig. 13a). To illustrate this two of these relations, problemSpec and numericalAlg, are shown in Fig. 14.^2^ The top-level call is shown in Fig. 15. Note that the construction of this top-level call can be done automatically from suitable meta-data describing valid Nektar++ parametrisations. For the purposes of this paper we have constructed the call by hand.

**Fig. 16:**
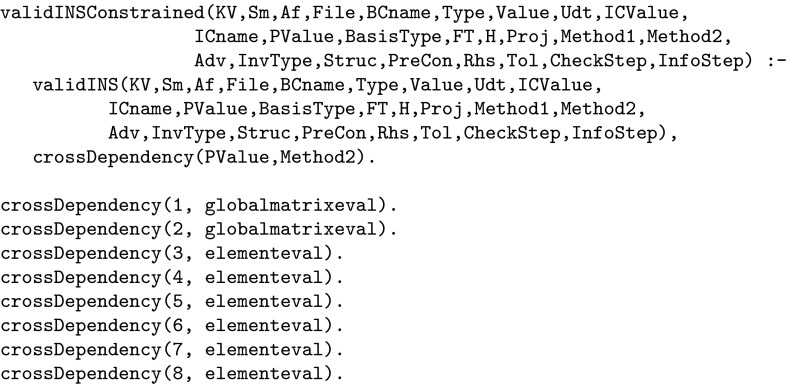
Top-level call for the Incompressible Navier–Stokes problem including the cross-dependency constraint

#### Constraining the Solver

We are now in a position to impose constraints on the various Nektar++ parameter settings. Referring back to the example given in the introduction, a constraint recommended by the developers of Nektar++, but not one that is imposed anywhere within the code, is to use a global matrix approach when the polynomial order is 1 or 2, and to use the elemental approach otherwise. The global matrix/elemental approaches are options within the Numerical Algorithm parameter set illustrated in Fig. 13a. This constraint introduces a cross-dependency between Problem Specification and Numerical Algorithm. Such cross-dependencies are common in HPC codes which is why the problem cannot be solved using simple parameter “trees”, as exemplified by Nekkloud.

The top-level call of the problem was given in Fig. 16. To include the dependency between the polynomial order and the numerical solution method, we introduce a new top-level call named validINSConstrained, which augments the original top-level call with the linking predicate, crossDependency which has the effect of tying polynomial orders 1 and 2 to the global matrix evaluation method and orders 3 upwards to the elemental method. This cross-dependency constraint is an example of “meta information” that is not captured at all in the application code. With this constraint in place it is now not possible to select incompatible values for the polynomial order and solution method. In this case the constraint captures explicitly the sort of information that is often confined to the user manual or the auspices of “received wisdom”.

## Related Work

Workflows are ubiquitous in many branches of computational science and engineering for coordinating distributed resources and services. Commonly-used systems include Taverna [13], which is a general-purpose framework supporting cross-language workflows, Kepler [15], which is targeted primarily towards bioinformatics pipelines and Cascading [1], which is a platform for developing workflow applications on top of Hadoop. The Python language has also been used as a workflow scripting language in PaPy [6], which is a lightweight toolkit for the specification of bioinformatics workflows. A detailed survey of various workflow systems, and an overview of the desirable features of workflow systems can be found in [3]. The specific issue of how workflows can facilitate the documentation of provenance of scientific output is surveyed in [10].

Many papers address the issue of workflow planning and optimization, for example [7, 11, 12], but the issue of semantic analysis of workflows for consistency has received rather less attention. Perhaps the closest work to our own is that of [4] which uses separate semantic annotations to determine whether two connected components within a workflow are semantically compatible; this is designed to augment the sort of semantic checking that can be achieved through traditional strong typing within workflows—see [14], for example.

## Conclusions

The realisation of a framework capable of capturing and effectively using the decisions inherent in any software development would have profound, beneficial, implications for the long term usability and sustainability of high performance codes. As we have seen above the methodology makes high performance applications accessible to and usable by end users who may not necessarily be conversant with the underlying methods and software used. However we also feel the methodology has further advantages when it comes to the long-term maintenance of complex codes. Systematic program modification would be facilitated. If changing circumstances require that a running application be modified the decision tree that led to the current state of the code could be accessed and traced back to the decision point(s) that are affected by the changed circumstance and a new code variant systematically derived by re-running the decision process with the new parameters. Provenance checking would also be facilitated. If it is required to archive the calculations leading to a published result it is necessary to archive both the input data and code used to produce these results. Archiving data is not an issue given adequate storage facilities. Archiving and reproducing the code is another matter. Code binaries may be stored but the machines and operating systems that supported them may change. With the framework described here it would only be necessary to store the abstract workflow used in any experiment. If provenance is needed to be tested the current, best, implementations of the abstract functions could be used and the newly instantiated workflow run on the archived data and the results compared. An elementary form of this provenance checking capability has already been implemented in our prototype system, but more work is needed.

In conclusion, we believe such a framework effectively supports the development, use, modification and sustainability of high performance codes in a manner that allows all members in the HPC Eco-system (users, method developers, infrastructure providers) to play their roles effectively, mediated by a structured methods and implementation repository.

## Footnotes

1 In practice it is probably preferable to establish name-spaces so that particular application workflows can be associated with a given Prolog database. In the current prototype the Prolog database is flat.

2 Notice that we make the simplifying assumption that the same solver (Method2) must be used by each of the backTransform, innerProduct, massMatrix and helmholtzMatrix steps. This is not imposed by Nektar++.
